# Immune-inflammatory biomarkers as prognostic factors for immunotherapy in pretreated advanced urinary tract cancer patients: an analysis of the Italian SAUL cohort

**DOI:** 10.1016/j.esmoop.2021.100118

**Published:** 2021-05-10

**Authors:** G. Fornarini, S.E. Rebuzzi, G.L. Banna, F. Calabrò, G. Scandurra, U. De Giorgi, C. Masini, C. Baldessari, E. Naglieri, C. Caserta, S. Manacorda, M. Maruzzo, M. Milella, C. Buttigliero, R. Tambaro, P. Ermacora, F. Morelli, F. Nolè, C. Astolfi, C.N. Sternberg

**Affiliations:** 1 Medical Oncology Unit 1, IRCCS Ospedale Policlinico San Martino, Genova, Italy; 2 Department of Internal Medicine and Medical Specialties, University of Genoa, Genoa, Italy; 3 Department of Oncology, Portsmouth Hospitals University NHS Trust, Portsmouth, UK; 4 Medical Oncology, Azienda Ospedaliera S. Camillo-Forlanini, Rome, Italy; 5 Medical Oncology, Azienda Ospedaliera Cannizzaro di Catania, Catania, Italy; 6 Medical Oncology, Istituto Scientifico Romagnolo per lo Studio e la Cura dei Tumori (IRST) – IRCCS, Meldola, Italy; 7 Medical Oncology, AUSL-IRCCS di Reggio Emilia, Reggio Emilia, Italy; 8 Oncology, Azienda Ospedaliero - Universitaria di Modena, Modena, Italy; 9 Division of Medical Oncology, IRCCS Istituto Tumori Bari Giovanni Paolo II - IRCCS, Bari, Italy; 10 Medical Oncology Unit, Azienda Ospedaliera S. Maria, Terni, Italy; 11 Medical Oncology, Azienda Ospedaliero-Universitaria Pisana, Pisa, Italy; 12 Medical Oncology Unit 1, Department of Oncology, Istituto Oncologico Veneto, IOV-IRCCS, Padua, Italy; 13 Dipartimento di Oncologia, Policlinico Universitario G.B. Rossi Borgo Roma, Verona, Italy; 14 Medical Oncology, Università degli Studi di Torino, Turin, Italy; 15 U.O.C di Oncologia Sperimentale Uroginecologica, I.N.T. IRCCS Fondazione G. Pascale, Naples, Italy; 16 Dipartimento di Oncologia, Azienda Ospedaliero Universitaria di Udine, Udine, Italy; 17 Casa Sollievo della Sofferenza, S. Giovanni Rotondo, Foggia, Italy; 18 IEO, Istituto Europeo di Oncologia IRCCS, Milan, Italy; 19 Medical Affairs & Clinical Operation, Roche S.p.A., Monza, Italy; 20 Hematology and Oncology, Englander Institute for Precision Medicine Weill Cornell Medicine, New York-Presbyterian, New York, USA

**Keywords:** biomarker, immunotherapy, PD-1, PD-L1, LDH, neutrophil-to-lymphocyte ratio (NLR), systemic immune-inflammation index (SII), immune-checkpoint inhibitor, prognostic, urothelial carcinoma

## Abstract

**Background:**

Reliable and affordable prognostic and predictive biomarkers for urothelial carcinoma treated with immunotherapy may allow patients' outcome stratification and drive therapeutic options. The SAUL trial investigated the safety and efficacy of atezolizumab in a real-world setting on 1004 patients with locally advanced or metastatic urothelial carcinoma who progressed to one to three prior systemic therapies.

**Patients and methods:**

Using the SAUL Italian cohort of 267 patients, we investigated the prognostic role of neutrophil-to-lymphocyte ratio (NLR) and systemic immune-inflammation index (SII) and the best performing one of these in combination with programmed death-ligand 1 (PD-L1) with or without lactate dehydrogenase (LDH). Previously reported cut-offs (NLR >3 and NLR >5; SII >1375) in addition to study-defined ones derived from receiver operating characteristic (ROC) analysis were used.

**Results:**

The cut-off values for NLR and SII by the ROC analysis were 3.65 (sensitivity 60.4; specificity 63.0) and 884 (sensitivity 64.4; specificity 67.5), respectively. The median overall survival (OS) was 14.7 months for NLR <3.65 [95% confidence interval (CI) 9.9-not reached (NR)] versus 6.0 months for NLR ≥3.65 (95% CI 3.9-9.4); 14.7 months for SII <884 (95% CI 10.6-NR) versus 6.0 months for SII ≥884 (95% CI 3.7-8.6). The combination of SII, PD-L1, and LDH stratified OS better than SII plus PD-L1 through better identification of patients with intermediate prognosis (77% versus 48%, respectively). Multivariate analyses confirmed significant correlations with OS and progression-free survival for both the SII + PD-L1 + LDH and SII + PD-L1 combinations.

**Conclusion:**

The combination of immune-inflammatory biomarkers based on SII, PD-L1, with or without LDH is a potentially useful and easy-to-assess prognostic tool deserving validation to identify patients who may benefit from immunotherapy alone or alternative therapies.

## Highlights

• Reliable biomarkers for immunotherapy may assist in treatment decision making and clinical trial design and interpretation.
• Immune-inflammatory biomarkers were investigated for their prognostic role within the Italian SAUL study cohort.
• ROC-based cut-offs were 3.65 for NLR and 884 for SII.
• Both NLR and SII were prognostic with SII performing slightly better than NLR.
• The combination of SII, PD-L1, and LDH stratified OS better than SII + PD-L1; both were independent prognostic factors.

## Background

The treatment landscape of locally advanced or metastatic urinary tract carcinoma has undergone minimal progress in the past decades and platinum-based chemotherapy regimens are still the standard of care for neoadjuvant, adjuvant, and first-line treatment.^1^ Recently, however, five new immunotherapeutic agents have become available. The anti-programmed cell death protein 1 (PD-1) antibodies pembrolizumab and nivolumab and anti-programmed death-ligand 1 (PD-L1) antibodies atezolizumab, durvalumab, and avelumab are currently the therapeutic options for second-line treatment of platinum-treated locally advanced or metastatic urothelial carcinoma (mUC).^2^^,^^3^ According to NCCN guidelines, atezolizumab and pembrolizumab are also first-line treatment options for PD-L1 positive, cisplatin-ineligible patients,^2^^,^^3^ regardless of PD-L1 expression in patients who are not eligible for any platinum-containing chemotherapy. Atezolizumab in combination with first-line chemotherapy demonstrated an advantage in progression-free survival (PFS), although with no significant difference in overall survival (OS), which was a co-primary endpoint.^4^^,^^5^ Recently, maintenance treatment with avelumab after first-line platinum-based chemotherapy showed a significantly prolonged OS advantage compared with chemotherapy alone for nonprogressive patients^6^ and is likely to become a new standard of treatment.

The Phase II IMvigor 210 trial investigated first-line atezolizumab for cisplatin-ineligible patients with mUC^7^ with an overall response rate (ORR) of 23% and median OS of 15.9 months. A second cohort of the same study included 310 patients who progressed after first-line platinum-based therapy, showing an ORR of 15% and a median OS of 7.9 months. Based on these results, the Food and Drug Administration (FDA) and European Medicines Agency (EMA) approved atezolizumab in both these settings. However, in the subsequent phase III IMvigor211 trial, atezolizumab did not meet the primary endpoint of OS compared with standard chemotherapy in patients who progressed to a platinum-containing regimen. The OS was investigated by a hierarchical statistical analysis aimed previously at the prespecified population of patients with tumors overexpressing PD-L1 or with at least 5% PD-L1 expression on tumor-infiltrating immune cells (ICs) (defined as IC2/3), who represented 25% of the overall study population.^8^ In the same setting, the phase III KEYNOTE-045 study reported a significant improvement in OS, but not in PFS, in favor of pembrolizumab versus chemotherapy in the overall study population of 542 patients unselected for PD-L1.^9^ Of note, in the subgroup analysis, the OS benefit from immunotherapy versus chemotherapy was significantly higher in patients with positive [defined as combined positive score (CPS) of positive tumor and ICs/total tumor cells ≥1%] or high (with a CPS ≥ 10%) PD-L1 tumors, but not in those with negative or low (CPS ≤ 10%) PD-L1 tumors.^9^ Similarly, a trend toward higher ORR, improved PFS, and OS has been observed with other anti-PD-1/PD-L1 agents in patients with PD-L1-positive mUC.^10^ However, different antibodies, types of cells assessed, and platforms for testing have led to inconsistencies among the different assays and their diagnostic and prognostic results.^11^ For these reasons, PD-L1 tumor expression alone cannot be currently considered as a reliable prognostic and/or predictive biomarker to select patients who are most likely to benefit from immunotherapy.

In addition to PD-L1, the use of inflammatory biomarkers, such as the neutrophil-to-lymphocyte ratio (NLR) and lactate dehydrogenase (LDH), has been explored.^12^^,^^13^ These biomarkers are known for their prognostic role in several tumor types, including genitourinary neoplasms. The interest in their use as prognostic biomarkers in tumors treated with immunotherapy has been recently increasing, particularly in melanoma and non-small-cell lung cancer.^12^^,^^14^, ^15^, ^16^, ^17^ In addition, the systemic immune-inflammation index (SII) combined with the monocyte-to-lymphocyte ratio has yielded promising results to identify poor responders to immune-checkpoint inhibitors (ICIs) in the mUC.^18^^,^^19^

The SAUL trial examined the safety and efficacy of atezolizumab in an international real-world setting on 1004 patients with locally advanced or mUC or nonurothelial urinary tract carcinoma who progressed to one to three prior systemic therapies.^20^ The efficacy observed in the overall study population and the IMvigor211-like subgroup of more selected patients was similar,^20^ despite the inclusion of special populations such as patients with Eastern Cooperative Oncology Group performance status (ECOG PS) 2, renal impairment, upper tract urothelial carcinoma or Bellini collecting duct tumors, autoimmune disease, brain disease, and HIV. This large real-life study population provides the opportunity to further investigate the role of potential biomarkers. Herein, we retrospectively investigated the prognostic role of (i) NLR and SII; and (ii) the best performing marker between NLR and SII in combination with PD-L1 with and without LDH in the Italian cohort of patients from the SAUL trial aiming at identifying a subset of patients who benefit the most from the immunotherapy.

## Materials and methods

### Study design

The SAUL study (NCT02928406) was a single-arm multicenter international open-label phase IIIB safety study of atezolizumab in locally advanced (T4b Nany or TAny N2–3) or metastatic (M1) measurable and/or nonmeasurable urothelial or nonurothelial carcinoma of the urinary tract (bladder, ureter, urethra, or renal pelvis).^20^ Patients with renal impairment, treated central nervous system metastases, or stable controlled autoimmune disease were eligible for enrollment. All participants must have had ECOG PS ≤2 and disease progression during or following one (subsequently amended to up to three) prior platinum- or non-platinum-based treatments (or intolerance if they had received two or more cycles) for inoperable, locally advanced, or metastatic disease. Patients received atezolizumab 1200 mg intravenously every 3 weeks until lack of clinical benefit, unacceptable toxicity, patient's or investigator's decision to discontinue therapy, or death. Assessments were carried out every 9 weeks for 12 months and then every 12 weeks. If patients discontinued atezolizumab, they were followed for 30 days after the last dose (or until initiation of another anticancer therapy if earlier).

### Study objectives

The primary objective was to explore the prognostic role of NLR and SII (defined as NLR × platelets) at baseline (i.e. within 7 days from the treatment start) in correlation with OS, PFS, and disease control rate (DCR; defined as the sum of complete or partial response, or stable disease for at least 4 weeks) in the Italian SAUL cohort. For this aim, we used (i) preplanned cut-offs as previously reported in the literature (NLR >3 and NLR >5; SII >1375); and (ii) study-defined cut-off values derived from the receiver operating characteristic (ROC) analysis based on the DCR. The secondary objective was the evaluation of the combination of the best performing biomarker between NLR and SII with PD-L1, with or without LDH, in relation to OS, PFS, and DCR. For this purpose, PD-L1 expression was rated in ICs as either low (0-1, expression on <5% of ICs) or high (2-3, PD-L1 expression on ≥5% of ICs) and LDH as above or below the upper limit of normal (ULN) as defined locally (≤ULN versus >ULN).

### Statistical analysis

All clinical data were analyzed by descriptive statistics, which was carried out using percentages for the binary variables, and mean and median for the continuous variables, reporting their respective dispersion values. For the comparison of percentages, means and medians, confidence limits and tests are provided, such as chi-square test or Fisher's test and Student's *t*-test or Wilcoxon test, as appropriate. The best thresholds for NLR and SII were derived using the ROC curve analysis based on the DCR.

Survival curves of OS and PFS were generated using the Kaplan–Meier method. Univariate differences in OS and PFS were evaluated using the log-rank test. Two-sided 95% confidence intervals (CIs) are provided for the main statistical estimators. Multivariate Cox regression analyses were carried out to determine the correlation between the inflammatory biomarkers and OS, PFS, and DCR. Results are reported as the hazard ratio or odds ratio, as appropriate, with the corresponding 95% CI. Regression models included terms for sex, age, ECOG PS, regional lymph nodes, creatinine clearance, and liver metastases, determined a priori based on the available literature.^21^ Statistical significance was defined as *P* < 0.05.

## Results

### Patient characteristics

Of the 1004 patients included in the SAUL study, 270 (27%) were enrolled in Italy. Three patients were excluded because they never started treatment; therefore, a total of 267 patients were included. Key demographic and clinical characteristics of this cohort are shown in Table 1. The median age was 69 years [interquartile range (IQR) 62-74], and most patients were male (82.8%). Almost all patients (97.9%) had distant metastasis at study entry and >70% had undergone previous treatment for cancer. At the data cut-off for primary analysis (16 September 2018), 114 (42.7%) were continuing treatment, while 153 (57.3%) had discontinued treatment. Of those discontinuing, 139 (90.9%) died, 10 (6.5%) were lost to follow-up, and 4 (2.6%) patients withdrew from the study. The median duration of follow-up was 9.5 months (95% CI 8.8-10.4). Median OS was 9.3 months (95% CI 6.7-10.9), median PFS was 2.2 months (95% CI 2.1-2.5), and the DCR was 37.8%.

**Table 1:** Patient demographics and clinical characteristics

Characteristic	*n* (%)
Age, years	
Median (IQR)	69 (62-74)
Sex	
Male	221 (82.8)
Female	46 (17.2)
ECOG	
0	144 (53.9)
1	109 (40.8)
2	14 (5.2)
PD-L1 expression	
IC0	57 (23.3)
IC1	110 (44.9)
IC2	56 (22.9)
IC3	22 (9.0)
Missing data	22 (8.3)
PD-L1 expression, grouped	
IC0-1	167 (68.2)
IC2-3	78 (31.8)
Histological type	
Urothelial	257 (96.2)
Nonurothelial	6 (2.3)
Mixed histology	4 (1.5)
Histological grade	
1	8 (3.1)
2	24 (9.4)
3	222 (87.4)
Missing data	13 (4.9)
Tumor location	
Bladder	203 (76.0)
Renal pelvis	27 (10.1)
Ureter	27 (10.1)
Urethra	3 (1.1)
Other	7 (2.6)
Regional lymph node at study entry	
N0	77 (28.8)
N1	56 (21.0)
N2	71 (26.6)
N3	47 (17.6)
Not applicable	16 (6.0)
Distant metastasis at study entry	
M0	8 (3.0)
M1	259 (97.0)
Liver metastasis at study entry	
No	188 (70.4)
Yes	79 (29.6)
Previous lines of cancer therapy	
0	78 (29.2)
1	143 (53.6)
2-3	46 (17.2)

ECOG, Eastern Cooperative Oncology Group; IC, immune cell; IQR, interquartile range; PD-L1, programmed cell death-ligand 1.

### Role of baseline NLR and SII

**Figure 1:**
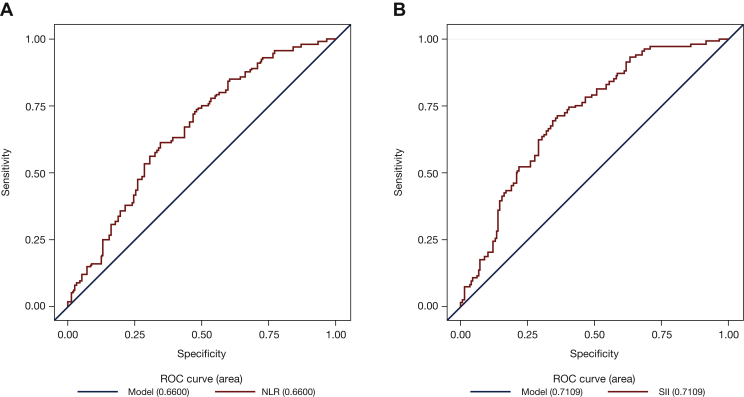
Receiver operating characteristic (ROC) analysis of (A) neutrophil-to-lymphocyte ratio (NLR) and (B) systemic immune-inflammation index (SII).

Data on baseline NLR and SII were available for 255 patients each (96%). The median NLR was 3.83 (IQR 2.67-5.78). The median SII was 948 (IQR 624.58-1574.75). ROC analysis of NLR and SII based on DCR defined cut-off values of 3.65 (sensitivity 60.4; specificity 63.0) and 884 (sensitivity 64.4; specificity 67.5), respectively (Figure 1); both performed better than the literature cut-offs in terms of discrimination of at-risk patients (Table 2). According to area under the curve (AUC) criteria, SII performed slightly better than NLR (AUC = 0.71 versus 0.66, respectively) to identify patients who experienced higher disease control (Figure 1). NLR <3.65 and SII <884 significantly predicted OS and PFS and both were associated with higher DCR (Table 2, Supplementary Figures S1 and S2, available at https://doi.org/10.1016/j.esmoop.2021.100118).

**Table 2:** Median PFS, OS, and DCR with hazard and odds ratios according to NLR and SII at different cut-offs, and to ECOG PS and liver metastases at study entry

Marker	Value	% Patients	PFS		OS		DCR	
			Median (95% CI), months	Adjusted HR^a^ (95% CI)	Median (95% CI), months	Adjusted HR^a^ (95% CI)	%	Adjusted OR^a^ (95% CI)
NLR	<3	32	4.0 (2.3-6.5)	1.00 (Ref)	NR	1.00 (Ref)	51.8	1.00 (Ref)
	≥3	68	2.1 (2.0-2.4)	1.50 (1.08-2.08)	7.9 (4.9-10.6)	1.40 (0.92-2.11)	33.9	0.54 (0.30-0.97)
	<5	69	2.9 (2.3-4.2)	1.00 (Ref)	13.5 (9.9-NR)	1.00 (Ref)	47.4	1.00 (Ref)
	≥5	31	2.0 (1.8-2.1)	1.37 (0.99-1.90)	3.6 (2.9-7.0)	2.05 (1.39-3.02)	22.5	0.34 (0.18-0.66)
	<3.65	46	3.8 (2.3-5.9)	1.00 (Ref)	14.7 (9.9-NR)	1.00 (Ref)	53.0	1.00 (Ref)
	≥3.65	54	2.1 (2.0-2.2)	1.43 (1.06-1.93)	6.0 (3.9-9.4)	1.61 (1.10-2.35)	28.3	0.39 (0.22-0.68)
SII	<1375	67	3.6 (2.3-4.3)	1.00 (Ref)	13.5 (9.9-NR)	1.00 (Ref)	49.7	1.00 (Ref)
	≥1375	33	2.0 (1.8-2.1)	1.91 (1.39-2.63)	3.6 (2.8-5.4)	2.45 (1.67-3.59)	19.1	0.22 (0.11-0.44)
	<884	44	4.2 (2.9-6.2)	1.00 (Ref)	14.7 (10.6-NR)	1.00 (Ref)	57.5	1.00 (Ref)
	≥884	56	2.1 (1.9-2.1)	1.79 (1.32-2.43)	6.0 (3.7-8.6)	1.99 (1.35-2.94)	25.3	0.25 (0.14-0.45)
ECOG PS	0	54	2.8 (2.3-4.2)	1.00 (Ref)	11.5 (9.9-NR)	1.00 (Ref)	47.9	1.00 (Ref)
	1-2	46	2.1 (1.9-2.2)	1.55 (1.16-2.07)	4.5 (3.2-7.0)	2.19 (1.52-3.16)	26.0	0.44 (0.24-0.82)
Liver metastasis	No	70	2.9 (2.2-4.2)	1.00 (Ref)	11.0 (9.3-NR)	1.00 (Ref)	46.3	1.00 (Ref)
	Yes	30	2.0 (1.7-2.1)	1.93 (1.41-2.63)	3.7 (2.5-4.9)	2.86 (1.98-4.13)	17.7	0.28 (0.14-0.56)

CI, confidence interval; DCR, disease control rate; ECOG PS, Eastern Cooperative Oncology Group performance status; HR, hazard ratio; mOS, overall survival; NLR, neutrophil-to-lymphocyte ratio; NR, not reached; OR, odds ratio; OS, overall survival; PFS, progression-free survival; Ref, reference; SII, systemic immune-inflammation index.

a HR and OR from multivariate Cox and logistic regression models, respectively.

In univariate analysis, OS according to NLR and SII with predetermined literature cut-offs and those found by ROC analysis were evaluated and the results are shown in Supplementary Figure S1, available at https://doi.org/10.1016/j.esmoop.2021.100118. All cut-off values gave significant differences in OS.

For the ROC-determined cut-off values, a significant difference was seen in median OS with NLR using a cut-off value 3.65 (log-rank test, *P* < 0.0001). Median OS was 14.7 months for NLR <3.65 [95% CI 9.9-not reached (NR)] compared with 6.0 for NLR ≥3.65 (95% CI 3.9-9.4). A significant difference was also seen in median OS with SII using a cut-off value of 884 (Log-rank test, *P* < 0.0001). Median OS was 14.7 months for SII <884 (95% CI 10.6 to NR) compared with 6.0 months for SII ≥884 (95% CI 3.7-8.6). Significant differences were also seen in PFS using the predetermined cut-offs and those found by ROC analysis for NLR and SII (Supplementary Figure S2, available at https://doi.org/10.1016/j.esmoop.2021.100118).

Multivariate Cox regression analyses adjusted for sex, age, PS, creatinine clearance, and hepatic and lymph node metastases confirmed that NLR and SII were prognostic factors for PFS, OS, and DCR independently of other covariates for all cut-off values (Table 2).

### Role of SII, PD-L1, and LDH

**Figure 2:**
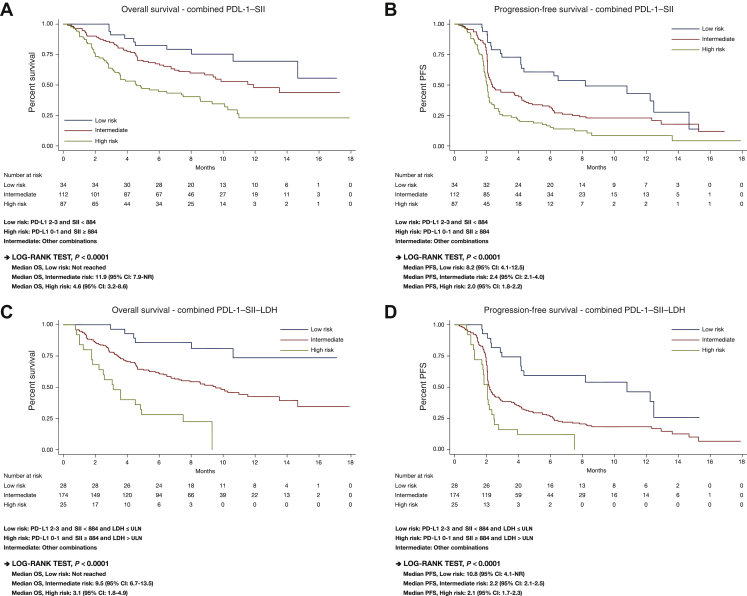
Overall survival (OS) and progression-free survival (PFS) according to PD-L1 + SII and PD-L1 + SII + LDH combinations. (A) OS, PD-L1 + SII; (B) PFS, PD-L1 + SII; (C) OS, PD-L1 + SII + LDH; (D) PFS, PD-L1 + SII + LDH. LDH, lactate dehydrogenase; NR, not reached; PD-L1, programmed cell death-ligand 1; SII, systemic immune-Inflammation index; ULN, upper limit of normal.

Because SII appeared to perform slightly better than NLR, in terms of both AUC by the ROC analysis (as mentioned above, Figure 1) and PFS and DCR prediction (Table 2), we next evaluated the combination of SII with PD-L1 with or without LDH. The combination of SII and PD-L1 with or without LDH identified three prognostic groups as low (PD-L1 IC 2-3, SII <884 with or without LDH ≤ ULN), high (PD-L1 IC 0-1, SII ≥884 with or without LDH >ULN), and intermediate (other combinations) risk which correlated significantly with PFS, OS, and DCR (Table 3 and Figure 2).

**Table 3:** Median PFS, OS, and DCR with hazard and odds ratios with PD-L1 + SII and PD-L1 + SII + LDH

Markers	Prognostic group	Patients, %	mPFS (months)	Adjusted hazard ratio (95% CI)	*P* value	mOS (months)	Adjusted hazard ratio (95% CI)	*P* value	DCR, %	Odds ratio (95% CI)	*P* value
PD-L1 + SII	Low	15	8.2	1 (Ref)		NR	1 (Ref)		73.5	1 (Ref)	
	Intermediate	48	2.4	1.70 (1.03-2.79)		11.9	1.62 (0.80-3.24)		44.6	0.33 (0.13-0.83)	
	Favorable	37	2	2.62 (1.55-4.44)	<0.0001	4.6	3.04 (1.50-6.19)	<0.0001	23	0.12 (0.04-0.33)	<0.001
PD-L1 + SII + LDH	Low	12	10.8	1 (Ref)		NR	1 (Ref)		75	1 (Ref)	
	Intermediate	77	2.2	2.18 (1.25-3.79)		9.5	2.90 (1.25-6.77)		38	0.22 (0.08-0.60)	
	Favorable	11	2.1	3.66 (1.85-7.23)	<0.0001	3.1	7.39 (2.83-19.31)	<0.0001	16	0.07 (0.02-0.31)	<0.001

CI, confidence interval; DCR, disease control rate; LDH, lactate dehydrogenase; mOS, median overall survival; mPFS, median progression-free survival; NR, not reached; OS, overall survival; PD-L1, programmed cell death-ligand 1; PFS, progression-free survival; Ref, reference; SII, systemic immune-inflammation index.

The combination of SII, PD-L1 and LDH seemed to be more accurate in the stratification of OS compared with the combination of SII with PD-L1 (Figure 2), through better identification of patients with intermediate prognosis (77% versus 48%, respectively) following immunotherapy (Table 3).

Multivariate analyses adjusted for sex, age, PS, creatinine clearance, and hepatic and lymph node metastases confirmed statistically significant correlations with OS and PFS for the SII + PD-L1 and SII + PD-L1 + LDH combinations, independently of other covariates (Supplementary Table S1, available at https://doi.org/10.1016/j.esmoop.2021.100118).

## Discussion

There is growing evidence regarding the use of inflammatory blood indices in patients with genitourinary cancer. Five meta-analyses^22^, ^23^, ^24^, ^25^, ^26^ indicated that pretreatment hematological indices, such as elevated NLR,^22^^,^^23^^,^^26^ platelet-to-lymphocyte ratio,^23^^,^^24^ and LDH,^25^ as well as decreased lymphocyte-to-monocyte ratio,^23^ are negative prognostic factors for UC patients. The prognostic role of NLR for advanced tumors was also confirmed by a meta-analysis of 66 studies on almost 25 000 patients.^27^ One study found that SII together with the monocyte-to-lymphocyte ratio predicted both disease progression and OS in UC patients prior to surgery.^18^

Regarding the role of these biomarkers in patients with UC undergoing treatment with ICIs, our group has recently explored the prognostic value of baseline NLR, with cut-offs ≥ 3 and ≥ 5, and of a urothelial immune prognostic index (UIPI) that was based on increased NLR and LDH.^12^ NLR and UIPI were significant predictors of PFS and OS with both having cut-offs of ≥ 3 and ≥ 5, respectively. Risk models combining inflammatory indices with clinical or genomic variables have been developed. A risk scoring using baseline platelet-to-lymphocyte ratio, presence of liver metastasis, albumin, and ECOG PS has been developed on a cohort of 67 UC patients treated with ICIs.^28^ A three-factor model including genomic (namely, a single-nucleotide variant count >9) and clinical (i.e. NLR <5 and lack of visceral metastasis) variables was related to benefit from ICI but not from taxane therapy in 62 patients with metastatic UC.^29^^,^^30^ In a recent large multicenter retrospective study on 463 pembrolizumab-treated patients with chemoresistant UC, a prognostic model based on ECOG PS, site of metastasis, hemoglobin levels, and the NLR was developed and internally validated.^31^ A five-factor prognostic model, based on pretreatment ECOG PS, presence of liver metastases, platelet count, NLR, and LDH has been validated within phase I/II clinical trials on 405 patients with metastatic UC treated with three PD-L1 inhibitors (i.e. atezolizumab, avelumab, and durvalumab) after platinum therapy.^32^ To date, this is the only externally validated risk model for immunotherapy in pretreated metastatic UC and also has a related web-based interactive tool to calculate the expected survival probability based on risk factors.^32^ These models provide useful and easy-to-obtain information for patient counseling and clinical trial design and interpretation.

The results of this study found that SII might be a better predictor of OS, PFS, and DCR than NLR in advanced pretreated UC tumors treated with immunotherapy, as it incorporates the platelet count and especially when combined with PD-L1 and LDH. Moreover, the triple combination of SII, PD-L1, and LDH performed better than SII plus PD-L1 in stratifying patients' outcomes by more accurately estimating patients with intermediate prognosis.

Our results on SII and NLR confirm the role of tumor inflammation in advanced UC, as reported for other tumors such as the non-small-cell lung cancer.^15^, ^16^, ^17^^,^^33^ In other solid tumors, a high SII has been reported to be an independent negative prognostic factor^34^, ^35^, ^36^ and NLR has been combined with PD-L1 and/or LDH.^33^^,^^37^ We have also recently examined changes in NLR and SII in patients with metastatic renal cell treated with nivolumab, finding that SII might correlate better than the NLR with survival outcomes.^38^^,^^39^ However, to our knowledge, SII has never been explored in combination with PD-L1 or LDH. In small-cell lung cancer, for example, Hong et al.^40^ reported that SII, LDH, stage, and response to therapy were all associated with OS, but the authors did not study a combination of these markers to assess if they performed better than each marker alone.

Other potential tissue biomarkers and clinical prognostic factors are under investigation in UC and other tumors. Tumor mutational burden (TMB) is another biomarker that has been explored to predict response to ICIs.^41^ The FDA approved the tumor-agnostic use of pembrolizumab for unresectable or metastatic solid tumors with high TMB (defined as ≥10 mutations/megabase).^42^ However, the phase II IMvigor 210 trial showed that TMB cut-off in mUC varied widely between the two cohorts of platinum-refractory and treatment-naïve cisplatin-ineligible patients and had low sensitivity.^43^ Further studies are, therefore, needed to validate the utility of these biomarkers in UC treated with immunotherapy.^44^^,^^45^ Using tissue microarray analysis, Li et al.^46^ found that UC could be classified as either immune high or low, with the former subgroup enriched in PD-L1 and with genomically unstable phenotype, which might make it more responsive to ICIs. A recent study has advocated the use of a score, namely the EPSILoN score, which combines three clinical and two inflammatory blood factors (i.e. smoking, ECOG PS, liver metastases, LDH, and NLR) to identify patients with advanced non-small-cell lung cancer who could likely benefit from second-line immunotherapy.^47^

The combination of immune-inflammatory biomarkers based on SII, PD-L1, with or without LDH identified herein is a potentially useful tool to identify patients who may benefit from immunotherapy. The results herein may suggest that immunotherapy alone should be the first option for patients with low risk, whereas for those with intermediate risk, immunotherapy is still an option but the participation in trials with investigational combination strategies might be favored; for patients with high risk, alternative options should be contemplated before considering immunotherapy. Our results further confirm that a combination of immune and inflammatory markers is better than any individual clinical or inflammatory blood factors, is easy to assess and routinely performed, as it does not require any special assays, and may be readily available. Other more sophisticated and currently not more accurate biomarkers, such as the TMB or gene signatures, are associated with substantial costs and longer turnaround times.^48^

A limitation of this study is that the PD-L1 results may apply only to the determination of its expression on ICs by the commercial immunohistochemical test used (Ventana SP142 PD-L1) and not to that quantified using the CPS, due to the aforesaid differences.^11^ Another limitation regards the lack of validation.

In summary, the combination of SII with PD-L1 with or without LDH may represent an easy-to-assess, cheap, and readily available prognostic tool for patients with metastatic urinary tract tumors who are candidates for immunotherapy to stratify their outcome and drive therapeutic decisions and deserves validation in larger cohorts including patients treated with chemotherapy to assess its predictivity, and in other treatment settings (e.g. first-line therapy). Furthermore, these immune-inflammatory factors might be explored in combination with other clinical prognostic factors to create more accurate predictive models.

## Ethics approval and consent to participate

The ethics committee at IRCCS Ospedale Policlinico San Martino, Genova, Italy was notified of the intent to carry out this retrospective data analysis.

## Consent for publication

Consent for publication was obtained from all authors.
