# Supplementary material for: Immune-inflammatory biomarkers as prognostic factors for immunotherapy in pretreated advanced urinary tract cancer patients: an analysis of the Italian SAUL cohort
Source: ESMO Open. 2021 May 10;6(3):100118. doi: 10.1016/j.esmoop.2021.100118 (PMC8134706; doi:10.1016/j.esmoop.2021.100118)

**Figure 1.** **ROC analysis of NLR (A) and SII (B).**


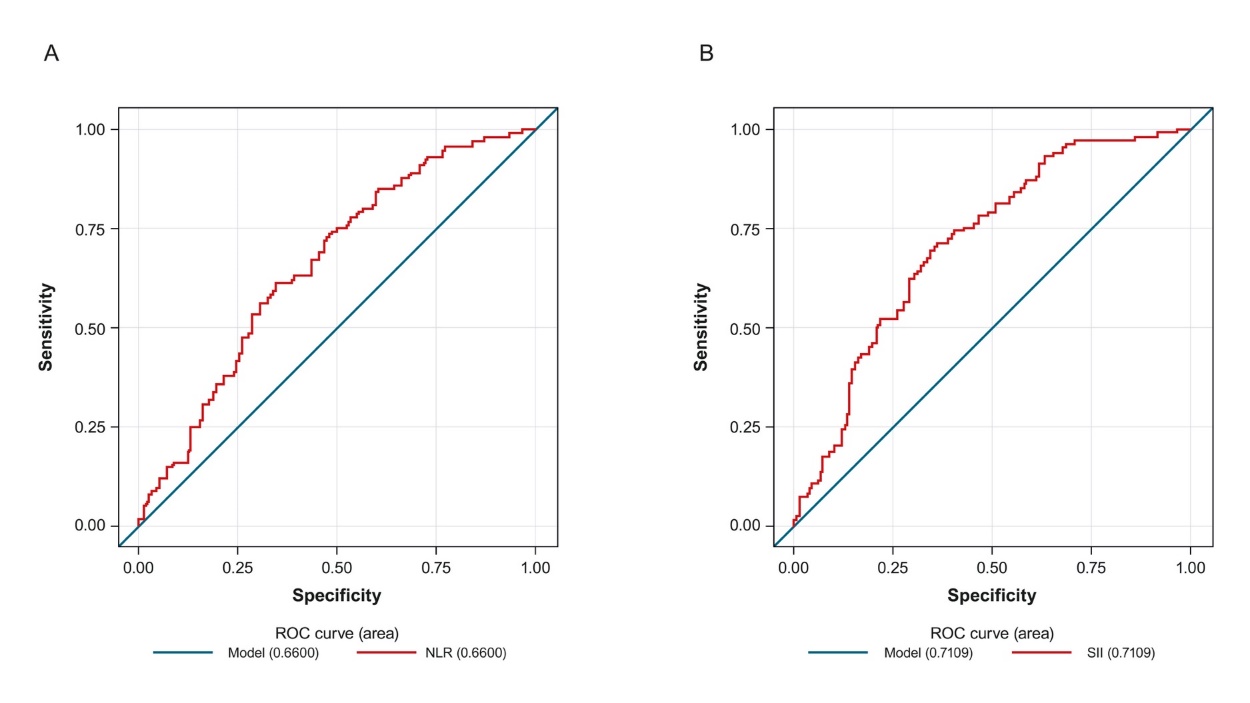


**Figure 2. OS and PFS according to PD-L1+SII and PD-L1+SII+LDH combinations. A) OS, PD-L1+SII; B) PFS, PD-L1+SII; C) OS, PD-L1+SII+LDH; D), PFS, PD-L1+SII+LDH.**


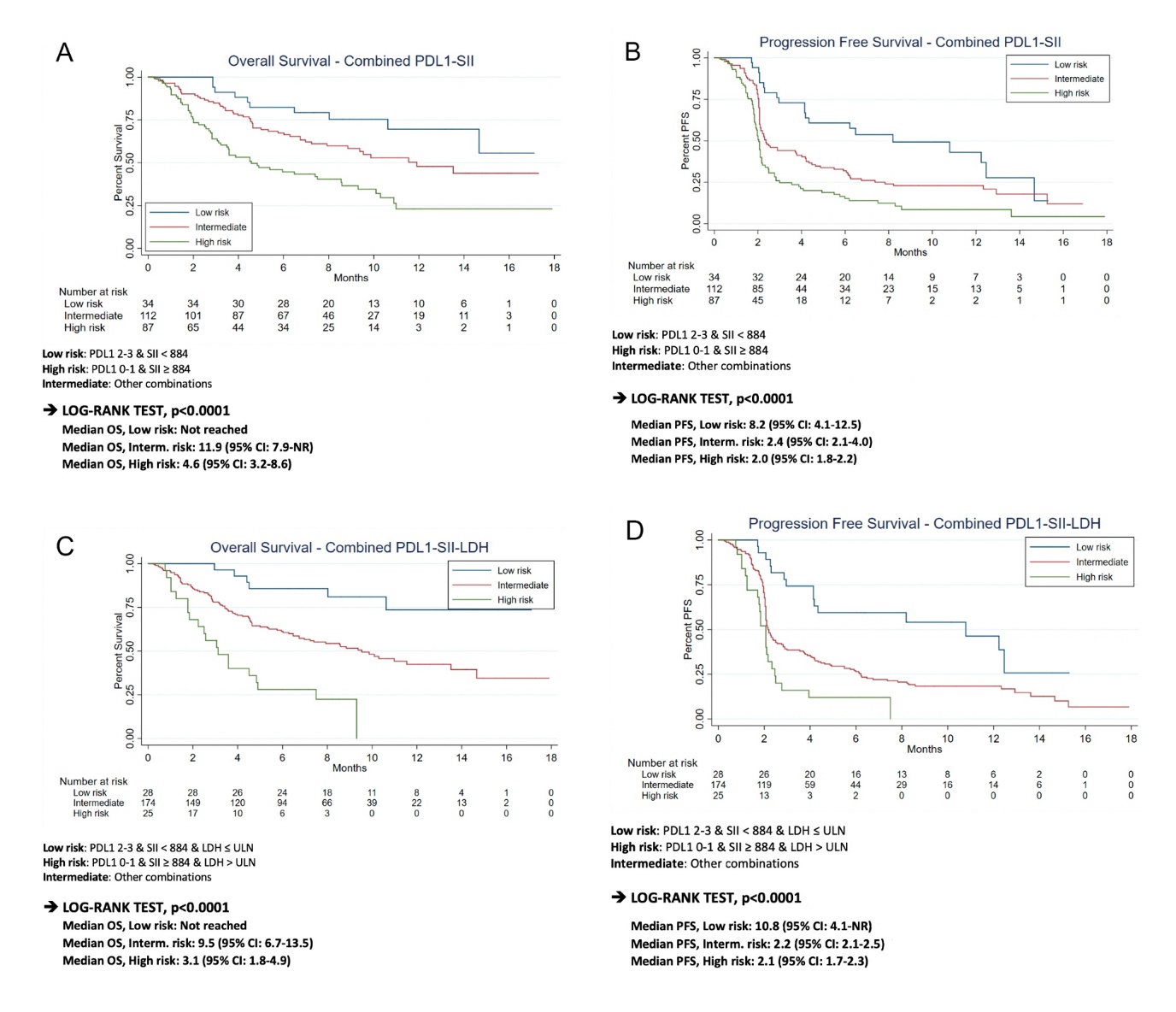


**Supplementary Figure 1**. **Overall survival according to NLR and SII with various cut-off values. A) NLR cut-off 3; B) NLR cut-off 5; C) NLR cut-off 3.65; D) SII cut-off 1375; E) SII cut-off 884.**


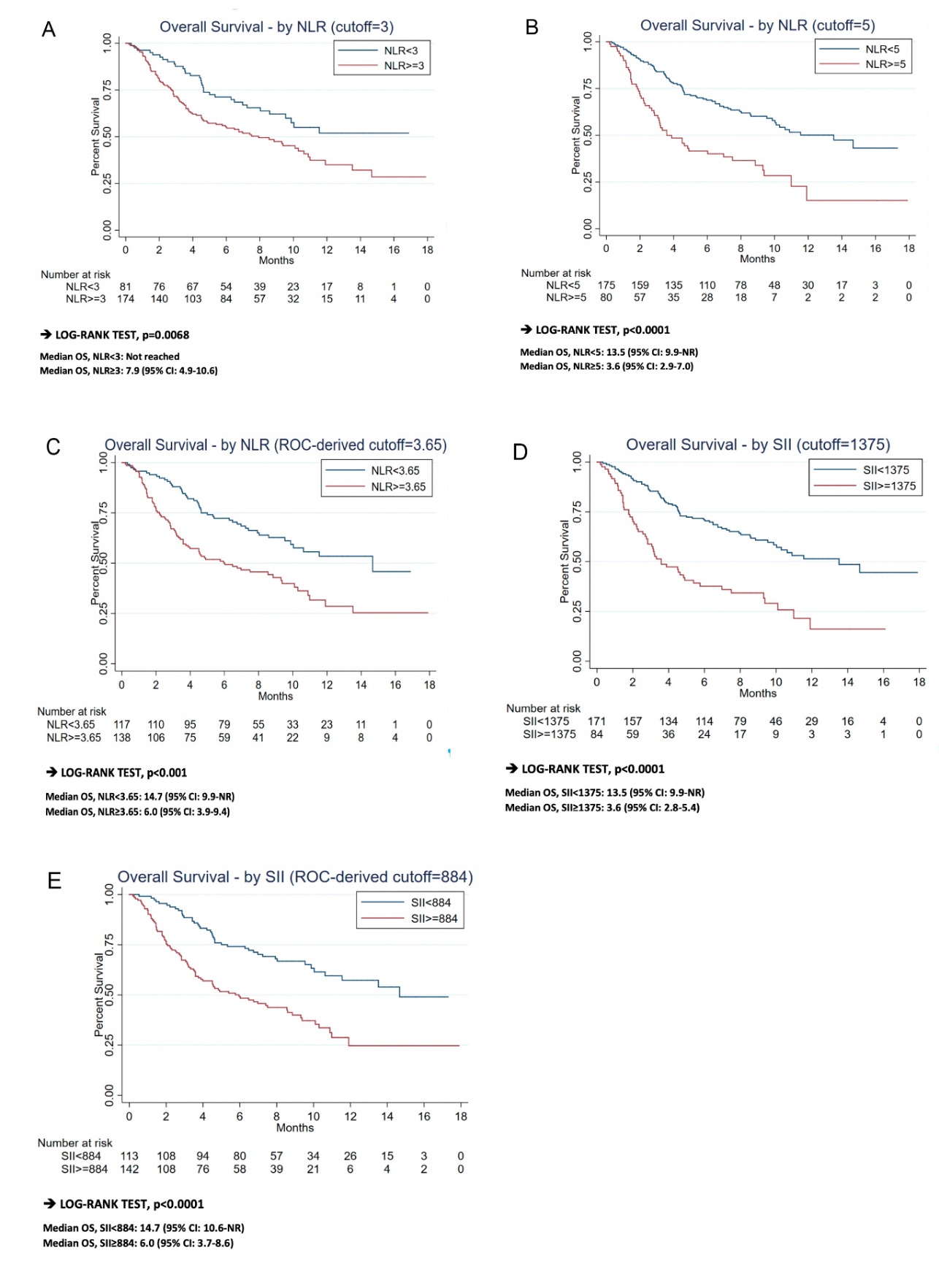


**Supplementary Figure 2**. Progression-free survival by NLR and SII with various cut-off values. A) NLR cut-off 3; B) NLR cut-off 5; C) NLR cut-off 3.65; D) SII cut-off 1375; E) SII cut-off 884.


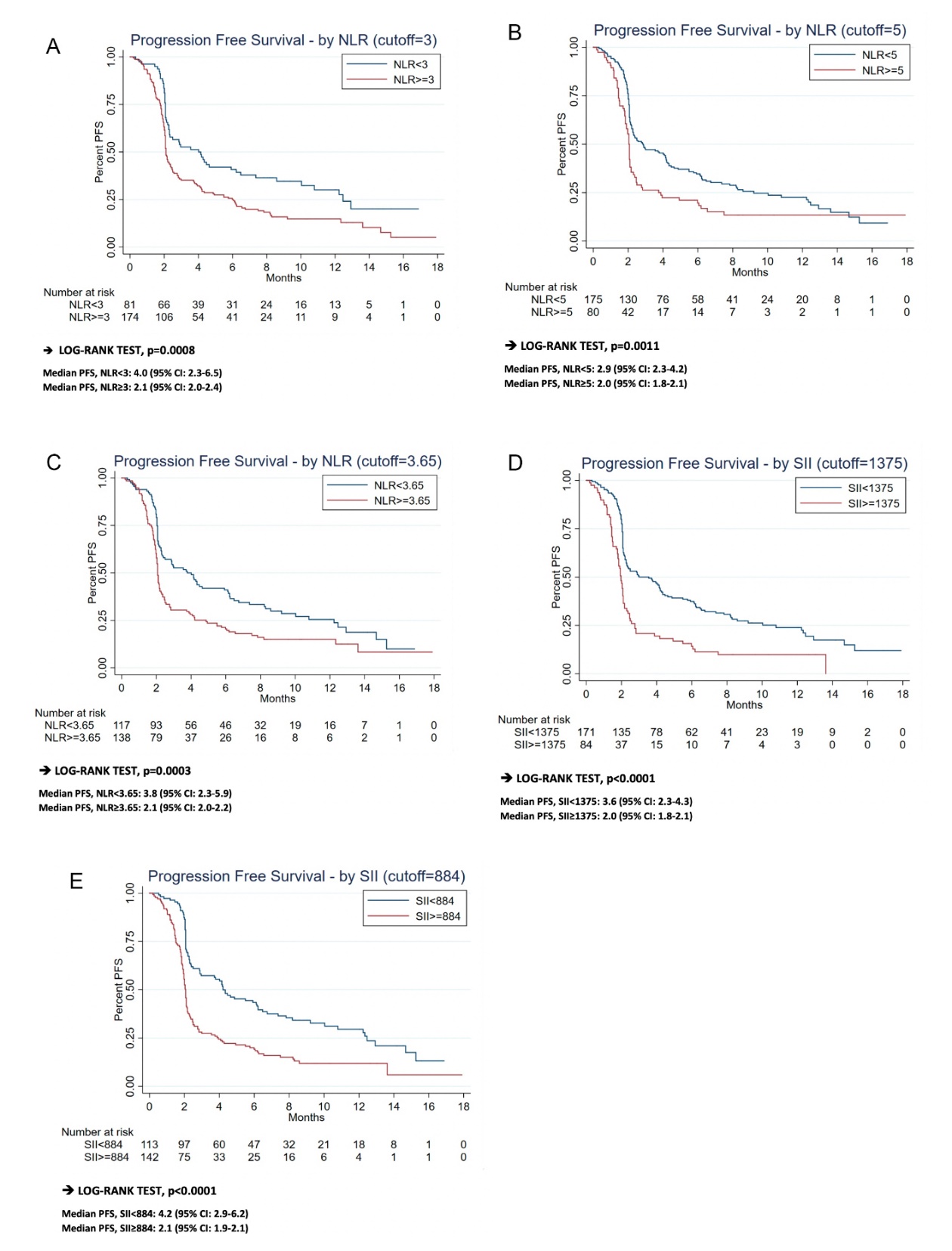

Supplement: Supplementary Figures S1 and S2 [file mmc1.docx]
