# Supplementary material for: Immune-inflammatory biomarkers as prognostic factors for immunotherapy in pretreated advanced urinary tract cancer patients: an analysis of the Italian SAUL cohort
Source: ESMO Open. 2021 May 10;6(3):100118. doi: 10.1016/j.esmoop.2021.100118 (PMC8134706; doi:10.1016/j.esmoop.2021.100118)
Supplement: Supplementary Table S1 [file mmc2.docx]

Supplementary Table 1. Multivariate hazard ratios (HR) of mortality and progression of disease, and corresponding 95% confidence intervals (CI), according to various risk profiles of patients.

| Combinations of factors | Overall Survival | Progression-free Survival |
| --- | --- | --- |
| PDL1 + SII | **Adjusted HR (95% CI)^a^** | |
| Low risk (PDL1 2-3 & SII<884) | 1 (reference) | 1 (reference) |
| Intermediate risk (Other combinations) | 1.62 (0.80-3.24) | 1.70 (1.03-2.79) |
| High risk (PDL1 0-1 & SII≥884) | 3.04 (1.50-6.19) | 2.62 (1.55-4.44) |
| *p-value for trend in risk* | *<0.001* | *<0.001* |
|  |  |  |
| PDL1 + SII + LDH |  |  |
| Low risk (PDL1 2-3 & SII<884 & LDH≤ULN) | 1 (reference) | 1 (reference) |
| Intermediate risk (Other combinations) | 2.90 (1.25-6.77) | 2.18 (1.25-3.79) |
| High risk (PDL1 0-1 & SII≥884 & LDH>ULN) | 7.39 (2.83-19.31) | 3.66 (1.85-7.23) |
| *p-value for trend in risk* | *<0.001* | *<0.001* |

^a^Computed using a multivariate Cox regression model, including terms for sex, age, ECOG, regional lymph nodes, creatinine clearance and liver metastases.
